# Levels of serum S100B are associated with cognitive dysfunction in patients with type 2 diabetes

**DOI:** 10.18632/aging.102873

**Published:** 2020-02-29

**Authors:** Huan Yu, Hui Li, Xiaoqian Liu, Xiaoli Du, Binbin Deng

**Affiliations:** 1 Department of Pediatrics, Tianjin Children’s Hospital, Beichen, Tianjin, PR China; 2 Department of Neurology, Harrison International Peace Hospital, Hengshui, Hebei, PR China; 3 Department of Pediatrics, The Fourth People’s Hospital of Hengshui, Hengshui, Hebei, PR China; 4 Department of Neurology, First Affiliated Hospital of Wenzhou Medical University, Wenzhou, PR China

**Keywords:** S100B, cognitive, T2DM, DACD

## Abstract

Objective: Previous studies have provided robust evidence that cognitive impairment exists in patients with type 2 diabetes. The predictive role of S100B in a variety of neurodegenerative diseases such as Alzheimer’s disease, has been shown to be closely related to cognitive function. The purpose of this study was to investigate the correlation between serum S100B levels and cognitive function in type 2 diabetes patients.

Results: The type 2 diabetes group scored lower than the healthy control group in all domains of cognitive function except language and attention, and the former group also had lower serum levels of S100B. Besides, serum S100B levels were lower in the type 2 diabetes patients with impaired cognition than in those with normal cognition. In addition, the moderate to severe cognitive impairment group had significantly lower levels than that in mild cognitive impairment group. After adjusting for confounding factors, serum S100B levels were positively correlated with cognitive function in type 2 diabetes patients.

Conclusions: Serum S100B levels were positively correlated with cognitive function in type 2 diabetes patients with cognitive impairment. It is suggested that S100B may be involved in the occurrence and development of cognitive dysfunction in type 2 diabetes patients and play a protective role.

Methods: The clinical data and biochemical indexes of ninety-six patients with type 2 diabetes and sixty-eight healthy subjects were collected. The levels of serum S100B were detected by enzyme-linked immunosorbent assay. Ninety-six type 2 diabetes patients were divided into a cognitive dysfunction group and a normal cognition group according to Mini-mental State Examination scores. To better understand the differences in various aspects of cognition, we used the Repeatable Battery for the Assessment of Neuropsychological Status scale for further evaluation. To study the relationship between serum S100B levels and cognitive impairment, the cognitive dysfunction group was divided into a mild cognitive impairment group and a moderate to severe cognitive impairment group for further study.

## INTRODUCTION

Type 2 diabetes mellitus (T2DM) is a metabolic disease characterized by chronic hyperglycemia. The acute and chronic complications can involve multiple organs, such as diabetic nephropathy and diabetic peripheral neuropathy, which are well described. However, the cognitive decline caused by diabetes has been less frequently mentioned [1]. To strengthen this concept, Mijnhout et al. proposed the concept of diabetes-associated cognitive decline (DACD) in 2006 [2]. Over time, DACD has increasingly become a hot issue in research. The main reason is the sharp rise in the number of people with diabetes worldwide. According to the latest data from the International Diabetes Federation (IDF) in 2017, there were approximately 451 million people with diabetes worldwide, and this number is expected to rise to 693 million by 2045 [3]. As that number grows, so does the number of people with cognitive impairment. Studies have shown that the risk of cognitive impairment in DM patients is 1.5 to 2.5 times higher than that in non-DM patients, and this risk significantly increases with age [4]. This will undoubtedly seriously affect people's quality of life. However, the mechanism is still not clear.

S100B is a member of the hand-type Ca^2+^ -binding protein family. It is expressed in astrocytes, mature oligodendrocytes, renal epithelial cells and other cells [5]. Intracellularly, S100B, as a calcium sensitive protein, regulates many activities such as signal transduction and interferes with cell proliferation, survival and differentiation [6]. Extracellularly, there is an increasing evidence showing that the measurements of S100B in biological fluids including peripheral blood can be used as a marker of cell injury in the nervous system [6–8]. Studies have shown that S100B is associated with cognitive function in many diseases, such as Alzheimer's disease(AD) [9, 10], cerebral vascular disease [11], schizophrenia and mood disorders [12]. However, the relationship between S100B levels and cognitive function in patients with T2DM is not clear, and this study was designed to investigate this relationship.

## RESULTS

Clinical data, biochemical indicators, S100B levels and RBANS scores were compared between the T2DM group and the healthy control group. The clinical data and biochemical indicators of the two groups are shown in Supplementary Table 1. The levels of FPG, HbA1c and TC in the T2DM group were significantly higher than those in healthy controls, and there was no significant difference in any of the other indexes. The serum S100B levels and RBANS scores of the two groups are shown in Supplementary Tables 2 and 3. We found that the T2DM group scored lower than the healthy control group in all domains of cognitive function except language and attention in the T2DM group were significantly lower than those in the healthy control group, and the former group also had lower serum levels of S100B.

Comparison of clinical data, biochemical indicators, S100B levels and RBANS scores between the normal cognition group and the cognitive dysfunction group. The clinical data and biochemical indicators of the patients in the two groups are shown in Table 1. The group with cognitive dysfunction was older and had higher total cholesterol (TC) and HAb1c levels (P<0.05) than the group with normal cognition. Other indexes showed no significant differences between the two groups. The serum S100B levels in the cognitive dysfunction group were lower than those in the normal cognition group (P<0.05; Table 2). The total score and subscores on the RBANS in cognitive dysfunction group were lower than those in normal cognition group (P<0.05; Table 3).

**Table 1:** Comparison of general information and biochemical indicators between two groups, *P<0.05 represents that the difference was statistically significant.

**Index**	**Normal cognitive (n=44)**	**Cognitive dysfunction(n=52)**	**t or Z or X^2^**	***P***
Age(years)	46.55±11.79	53.02±10.06	-2.092	0.005*
Education(years)	12.07±3.20	11.12±2.90	5.732	0.677
Sex(man/woman)	24/20	30/22	0.96	0.837
BMI(kg/m^2^)	25.71±3.20	24. 26±4.70	3.856	0.050
TC(mmol/L)	4.73±0.92	5.18±0.98	-2.336	0.022*
TG(mmol/L)	2.44±1.41	2.09±1.21	1.983	0.159
HDL(mmol/L)	1.00±0.35	0.99±0.23	0.120	0.730
LDL(mmol/L)	2.44±1.41	2.44±1.41	-0.661	0.510
FPG(mmol/L)	8.44±2.53	8.84±4.5	0.458	0.499
HbA1c(%)	6.95±6.7	8.33±8.65	5.386	0.020*
Course(years)	4.71±4.38	6.78±6.6	2.016	0.156

**Table 2:** Comparison of serum S100B level between two groups, *P<0.05 represents that the difference was statistically significant.

**Index**	**Normal cognitive (n=44)**	**Cognitive dysfunction (n=52)**	**t**	***P***
S100B(ug/L)	0.164±0.029	0.117±0.032	7.348	0.000*

**Table 3:** Comparison of cognitive status between two groups, *P<0.05 represents that the difference was statistically significant.

**Index**	**Normal cognitive(n=44)**	**Cognitive dysfunction(n=52)**	**T or Z**	***P***
Immediate memory	95.02±14.54	76.58±15.70	26.218	0.000*
Visuospatial	94.75±16.00	79.15±12.07	19.538	0.000*
Language	103.84±9.76	97.62±7.61	3.508	0.001*
Attention	109.91±11.16	94.87±17.87	23.265	0.000*
Delayed memory	100.65±8.52	89.02±11.16	28.228	0.000*
Total score	102.14±10.94	82.48±8.94	9.685	0.000*

Comparison of clinical data, biochemical indicators, S100B levels and RBANS scores between the mild cognitive impairment group and the moderate to severe cognitive impairment group. The moderate to severe cognitive impairment group had higher FPG and HAb1c levels than the mild cognitive impairment group, as shown in Table 4 (P<0.05). Other indexes showed no significant differences between the two groups. The serum S100B levels in the moderate to severe cognitive impairment group were lower than those in mild cognitive impairment group (P<0.05; Table 5). The total score and subscores on the RBANS are shown in Table 6. Not only the total scores, but also the immediate memory, visuospatial, attention and delayed memory scores were significantly lower in the moderate to severe cognitive impairment group than in the mild cognitive impairment group (P<0.05).

**Table 4:** Comparison of general information and biochemical indexes between mild cognitive impairment and moderate-severe cognitive impairment group, *P<0.05 represents that the difference was statistically significant.

**Index**	**Mild cognitive impairment (n=34)**	**Moderate-severe cognitive impairment (n=18)**	**t or Z or X^2^**	***P***
Age(years)	52.91±10.42	53.22±9.64	-0.105	0.917
Education(years)	11.14±3.16	11.05±2.41	3.668	0.817
Sex(man/woman)	20/14	10/8	0.51	1.00
BMI(kg/m^2^)	24.67±3.98	23.49±5.87	0.063	0.802
TC(mmol/L)	5.31±1.09	4.94±0.71	1.271	0.210
TG(mmol/L)	2.11±1.08	2.05±1.46	0.577	0.447
HDL(mmol/L)	0.97±0.24	1.02±0.21	-0.837	0.406
LDL(mmol/L)	3.23±1.08	3.16±0.80	0.221	0.826
FPG(mmol/L)	7.32±2.86	11.7±5.63	-3.091	0.005*
HbA1c(%)	7.61±2.74	9.7±2.87	-2.569	0.013*
Course(years)	6.44±6.39	7.42±7.11	0.369	0.544

**Table 5:** Comparison of serum S100B level between mild cognitive impairment and moderate-severe cognitive impairment group, *P<0.05 represents that the difference was statistically significant.

**Index**	**Mild cognitive impairment (n=34)**	**Moderate-severe cognitive impairment (n=18)**	**t**	***P***
S100B(ug/L)	0.132±0.027	0.087±0.015	7.579	0.000*

**Table 6:** Comparison of cognitive status between mild cognitive impairment and moderate-severe cognitive impairment group, *P<0.05 represents that the difference was statistically significant.

**Index**	**Mild cognitive impairment (n=34)**	**Moderate-severe cognitive impairment(n=18)**	**t or Z**	***P***
Immediate memory	81.82±14.62	66.66±12.89	10.443	0.001*
Visuospatial	83.58±10.19	70.77±11.03	11.675	0.001*
Language	98.50±7.36	95.94±8.01	1.155	0.253
Attention	102.50±8.56	80.44±21.90	23.408	0.000*
Delayed memory	93.26±7.27	81.00±12.92	3.724	0.001*
Total score	87.05±5.36	73.83±7.93	7.138	0.000*

Correlation between S100B levels and clinical data, biochemical indicators, RBANS scores in the cognitive dysfunction group. Our statistical analyses showed that serum S100B in cognitive dysfunction group were negatively correlated with the course of the disease (r =-0.277, P =0.047; Table 7) and positively correlated with visuospatial (r =0.287, P =0.039; Table 8), attentional (r =0.469, P<0.001, Table 8) and total RBANS(r =0.334, P = 0.016; Table 8)scores. After adjusting for confounding factors including age, sex, education, BMI, FPG, HbA1C, TC, TG, HDL and LDL, the correlations between serum S100B levels and the course of the disease (R^2^ =0.235, P<0.001; Table 9) and total RBANS scores remained the same (R^2^ =0.341, P<0.001; Table 9).

**Table 7:** The relationship of serum S100B with clinical indexes in cognitive dysfunction group, *P<0.05 represents that the difference was statistically significant.

**Index**	**S100B**	
	**r**	**P**
FPG	-0.207	0.141
HbA1C	-0.103	0.467
course	-0.277	0.047*

**Table 8:** The relationship between serum S100B and cognitive status, *P<0.05 represents that the difference was statistically significant.

**Index**	**S100B**	
	**r**	***P***
Immediate memory	0.191	0.176
Visuospatial	0.287	0.039*
Language	0.129	0.364
Attention	0.469	0.000*
Delayed memory	0.262	0.061
Total score	0.334	0.016*

**Table 9:** The result of stepwise regression, *P<0.05 represents that the difference was statistically significant.

**Index**	**S100B**		
	**R^2^**	***P***	**F**
Total score	0.235	0.000*	16.705
course	0.341	0.000*	14.196
Visuospatial	—	0.844	—
Attention	—	0.272	—

## DISCUSSION

DACD has received increasing attention from researchers. Since more than 90% of diabetes cases are T2DM, we designed the present study to focus on this population. To the best of our knowledge, this is the first study to examine the correlation between serum S100B and cognitive function in T2DM patients. The current literature identifies the following possible mechanisms for DACD: 1) High glucose toxicity: high blood glucose affects the functions of neurons, axons and synapses in the hippocampal CA1 region, changes the plasticity of synapses, leads to a decrease in long-term potentiation (LTP), and affects the process of learning and memory [13]. High glucose toxicity can also activate the hexosamine and polyol pathways, leading to oxidative stress and increased advanced glycation end products, which, in turn, cause neurodegeneration [14]. 2) Insulin resistance and insulin deficiency: insulin resistance leads to abnormal islet signal transduction, tau hyperphosphorylation, and neurodegeneration [15]. Studies have shown that insulin has neurotrophic effects [16], and insulin-deficient diabetic mice exhibit learning disabilities associated with impaired insulin signaling and increased GSK3 activity in the brain [17]. Intranasal insulin administration has been shown to improve the cognitive function of healthy people and people with cognitive impairment, which validates the neurotrophic effect of insulin [18]. In addition, DACD has been linked to hypoglycemia [19], inflammation and other factors [20, 21].

Our study found that serum S100B levels were positively correlated with cognitive function in T2DM patients with cognitive impairment, which suggests that S100B may be involved in the emergence and progression of cognitive dysfunction in T2DM. S100B, as a member of the calcium-binding protein family, is mainly expressed in astrocytes and plays important roles both intracellularly and extracellularly. Intracellularly, this protein can transfer signals and intervene in cell proliferation, survival and differentiation [22, 23]. Extracellularly, the effects depend on the concentration [24]. At micromolar concentrations, S100B may foster inflammatory effects counteracting neuroplasticity and induce apoptotic neuronal death [25, 26]. At nanomolar concentrations, S100B has been indicated to have neurotrophic activity [24]. Previous studies have shown that the hippocampus is an important cognitive region of the brain, and cognitive dysfunction in diseases such as DM and AD has been found to be related to the hippocampus [27, 28]. Nanomolar concentrations of S100B can promote hippocampal progenitor cell proliferation, neuronal differentiation and cognitive recovery [26]. This protein can also protect hippocampal neurons against excitotoxic injury [29].

In addition, S100B antiserum blocks LTP in hippocampal slices and promotes memory deficits in various tasks in a rat model [30, 31]. These effects may be connected to the survival and differentiation of nerve cells and the neurochemical processes related to learning and memory [32]. Therefore, we speculate that S100B exerts a neuroprotective effect against cognitive impairment in T2DM. The possible mechanisms of this effect are as follows: 1) The neuroprotective effect of S100B in T2DM may be related to the upregulated expression of the receptor for advanced glycation end products (RAGE). At low levels, binding between S100B and its receptor RAGE activates the Ras-MEK-ERK1/2-NF-κB pathway, which upregulates the expression of Bcl-2 and plays an antiapoptotic role [5]. 2) Nanomolar concentrations of extracellular S100B can protect hippocampal neurons against glutamate-induced injury [33]. Studies have shown that there is a decrease in glutamate uptake in the hippocampus of rats with STZ-induced diabetes [28, 34]. The impairment of glutamate uptake results in elevated extracellular levels of glutamate and leads to excitotoxicity. Accordingly, the GluN1 subunit of the NMDA receptor, which is downregulated by chronic excitotoxicity [35], was found to be reduced in STZ-diabetic animals [28, 34]. The combination of excitotoxicity and alterations in glutamate receptor expression can cause glutamatergic dysfunction and eventually lead to cognitive deficits in DM [36]. S100B can protect hippocampal neurons against NMDA-mediated glutamate toxicity [29]. In addition, research by Francine Tramontina et al. showed that glutamate uptake was stimulated by S100B and decreased in the presence of anti-S100B, which suggests that extracellular S100B stimulation of glutamate uptake by hippocampal astrocytes may be involved in its neuroprotective effect [32].

Our study found that serum S100B was significantly lower in T2DM patients than in healthy controls, which was consistent with the findings of Asuman Celikbilek and Hovsepyan MR [37, 38]. In addition, Nardin P et al. observed the same significant decrease in S100B in the serum of rats with STZ-induced diabetes [28]. Recently, Leticia Rodrigues et al. observed fresh hippocampal slices of STZ-treated mice and found that the level of S100B protein decreased as glucose metabolism changed [39]. Another study showed that in healthy people, peripheral blood glucose/insulin increases, accompanied by a decrease in S100B levels [40]. These results indicate that glucose metabolism affects the level of S100B. The exact mechanism of the effect is not clear. The following points may be relevant: 1) T2DM is characterized by chronic hyperglycemia, which may lead to decreased secretion of S100B from astrocytes and Schwann cells [41]. 2) Research has shown that insulin positively modulates S100B secretion in hippocampal slices [42], while antipsychotics appear to decrease insulin sensitivity [43] as well as S100B secretion [44, 45]. Moreover, antidepressants that increase S100B secretion, such as fluoxetine, seem to increase insulin sensitivity [46, 47]. In summary, the decrease in S100B in patients with T2DM may be related to insulin resistance and deficiency. In addition, we found that the levels of serum S100B in patients with cognitive impairment in T2DM were negatively correlated with the progression of the disease, suggesting that S100B may undergo changes during the emergence and progression of cognitive impairment in T2DM.

However, the cognitive implications of S100B are not limited to T2DM: studies have shown that this protein is involved in cognitive impairment in a variety of diseases, including cerebral small vessel disease and AD [9, 48]. Cerebral small vessel disease (CSVD) is an age-related disease affecting the small blood vessels of the brain. This condition is the most common cause of vascular dementia [49]. Furthermore, CSVD is a frequent comorbidity of T2DM [50]. This co-occurrence can be a cofounding factor for our research. To avoid this effect, one of the authors, Li Hui from the Department of Neurology, conducted voluntary head MRI assessments of diabetic patients, excluding those who had any of the following six injuries were enrolled: recent small subcortical infarcts, lacunae of presumed vascular origin, white matter hyperintensities of presumed vascular origin, perivascular space, cerebral microbleeds and brain atrophy [51]. Thus, the potential interference of CSVD was excluded from that study. The pathophysiological processes of AD are similar to those observed in T2DM [52, 53]. There is substantial evidence suggesting that early AD patients have elevated S100B levels, leading to cognitive impairment [9]. Peskind et al. found that S100B synthesis and secretion may be normal or decreased at the end of the course of AD [54]. Yardan et al. suggested that the abundance and distribution of S100B as well as its putative role in the onset and progression of AD could notionally change over the course of the disease. Those researchers proposed that chronically suppressed levels of extracellular S100B might be detrimental to neuronal function and be implicated in AD onset [55]. Our conclusion suggests that S100B may play a neuroprotective role against the emergence and progression of cognitive impairment in T2DM. The different roles of S100B in AD and type 2 diabetes may be related to disease progression and different disease stages. Further clinical and animal research is needed on this topic.

## MATERIALS AND METHODS

### Subjects

This study included sixty-eight healthy subjects and ninety-six outpatients from The First Affiliated Hospital of Wenzhou Medical University who met the diagnostic criteria for diabetes from the World Health Organization 1999. All enrolled patients has been receiving oral hypoglycemic drugs or routine insulin therapy. The exclusion criteria were as follows: 1) patients with recent trauma or major surgery; 2) patients with heart diseases, such as myocardial infarction; 3) patients with severe mental disorders, such as schizophrenia or depression; 4) patients with cancer; 5) patients with known diseases that affect cognitive function, including neurodegenerative diseases, diabetes retinopathy, diabetic kidney disease, and vascular dementia (Brain MRI was done voluntarily to rule out vascular factors); 6) patients with other endocrine and metabolic diseases, such as thyroid diseases; and 7) Other neurological diseases, such as epilepsy or multiple sclerosis. All subjects voluntarily participated in this study and signed the informed consent form. Our research was also approved by the ethics committee.

### Grouping and clinical data collection

The ninety-six patients were divided into a normal cognition group and a cognitive dysfunction group, and the cognitive dysfunction group was further divided into mild and moderate to severe groups according to Mini-mental State Examination (MMSE) scores. Clinical data and biochemical indicators of the participants were collected, including age, gender, course of the disease, education level, BMI, as well as fasting blood glucose (FPG), glycosylated hemoglobin (HbA1c), and blood lipids. All biochemical indexes were tested by the biochemical laboratory of our hospital, and an official report was issued.

### Cognitive function assessments

The MMSE is the preferred scale for dementia screening. The scale included seven dimensions: time orientation, location orientation, immediate memory, attention and computation, delayed memory, language and visual space. There were 30 questions in total, with 1 point for each correct answer and 0 points for wrong or unknown answers. The total score could range from 0 to 30 points. And we further grouped the cognitive impairment patients according to the following criteria: MMSE score ≥27 indicated normal cognition, 21-26 indicated mild cognitive impairment, 10-20 indicated moderate cognitive impairment, and less than 10 indicated severe cognitive impairment.

The Repeatable Battery for the Assessment of Neuropsychological Status (RBANS) contained 12 tests and five scores were calculated after adjustment for age to assess cognition in five different aspects of function: immediate memory (including story memory and list learning subtests), visuospatial (including line orientation and figure copy subtests), language (including semantic fluency and picture naming subtests), attention (including coding and digit span subtests), and delayed memory (including figure recall, story recall, list recall and recognition subtests). All trials were conducted by two uniformly trained clinicians, and the correlative coefficient for MMSE (or RBANS) total scores between the two clinicians was 0.8.

### Blood samples for detection of S100B levels

All participants were subjected to fasting venous blood extraction of 5 ml from 8 am to 9 am on the day of the cognitive function testing. The samples were subsequently centrifuged for 15 min at 3500 rpm at 4 °C. Serum S100B levels were determined by a commercially available ELISA kit (Cosmo Bio, Japan) with an interassay coefficient of variance of 5–9%. The sensitivity and dynamic range of the S100B assay is 0.06–0.53 ug/L.

### Data analysis

All variables are expressed as the mean ± standard deviation. T tests and chi-square tests were used to compare the mean values of the variables among groups, and Pearson bivariate correlation analysis was used to study the correlations between clinical data, biochemical indexes and RBANS scores. Stepwise regression analysis was used to evaluate the combined effects of potential confounding variables on serum S100B levels and RBANS scores. P<0.05 was considered statistically significant. SPSS 24.0 software was used to perform the statistical analysis.

## Supplementary Material

Supplementary Tables
