# Supplementary material for: Levels of serum S100B are associated with cognitive dysfunction in patients with type 2 diabetes
Source: Aging (Albany NY). 2020 Feb 29;12(5):4193–203. doi: 10.18632/aging.102873 (PMC7093188; doi:10.18632/aging.102873)
Supplement: Supplementary Tables [file aging-12-102873-s001..pdf]

SUPPLEMENTARY TABLES

Supplementary Table 1. Comparision of clinical data and biochemical indicators between T2DM and health controls, **P*<0.05 represents that the difference was statistically significant.

Index	T2DM (n=96)	Controls (n=68)	t or Z or X^2	*P*
Age(years)	50.05±11.31	48.41±12.13	0.888	0.376
Education(years)	11.55±3.07	11.12±2.76	5.732	0.677
Sex(man/woman)	54/42	29/39	2.947	0.086
BMI(kg/m^2)	24.93±4.13	24.87±3.05	0.091	0.927
TC(mmol/L)	4.98±0.98	4.64±1.00	2.129	0.035*
TG(mmol/L)	2.26±1.31	1.88±1.84	1.530	0.128
HDL(mmol/L)	1.00±0.29	1.07±0.23	-1.713	0.089
LDL(mmol/L)	3.16±0.88	3.04±0.65	0.922	0.358
FPG(mmol/L)	8.41±3.18	4.85±0.46	10.799	0.000*
HbA1c(%)	7.74±2.83	4.80±0.54	9.952	0.000*
Course(years)	5.83±5.76	—	—	—

Supplementary Table 2. Comparision of serum S100B levels between T2DM and health controls, **P*<0.05 represents that the difference was statistically significant.

Index	T2DM (n=96)	Controls (n=68)	t	*P*
S100B(ug/L)	0.139±0.039	0.344±0.086	-18.452	0.000*

Supplementary Table 3. Comparision of cognitive status between T2DM and health controls, **P*<0.05 represents that the difference was statistically significant.

Index	T2DM (n=96)	Controls (n=68)	T or Z	*P*
Immediate memory	85.03±17.71	91.56±16.76	-2.378	0.019*
Visuospatial	86.30±15.78	93.21±13.06	-2.935	0.004*
Language	100.47±9.16	101.25±9.09	-0.540	0.590
Attention	101.76±16.88	102.51±16.81	-0.282	0.778
Delayed memory	94.07±11.42	97.590.46	-2.010	0.046*
Total score	91.49±13.93	95.88±12.76	-2.059	0.041*
